# Gambogic acid affects ESCC progression through regulation of PI3K/AKT/mTOR signal pathway

**DOI:** 10.7150/jca.41115

**Published:** 2020-07-20

**Authors:** Jiarui Yu, Wei Wang, Weinan Yao, Zhao Yang, Peng Gao, Meiyue Liu, Huan Wang, Siyuan Chen, Dan Wang, weixi Wang, Guogui Sun

**Affiliations:** 1 School of Clinical Medicine, Affiliated Hospital, School of Public Health, North China University of Science and Technology, Tangshan, Hebei 063000, China.; 2 Department of Radiation Oncology, North China University of Science and Technology Affiliated People's Hospital, Tangshan, Hebei 063000, China.

**Keywords:** ESCC, Gambogic acid, Proliferation, Apoptosis, PI3K/AKT/mTOR

## Abstract

Esophageal squamous cell carcinoma (ESCC) is an invasive gastrointestinal malignancy and in urgent need of new effective therapies. Gambogic acid (GA) exhibits anti-cancer effects in many cancer cells, but it remains to be determined whether GA has the same effect on ESCC. Here, we reported that GA treatment caused an inhibition in ESCC cell proliferation, migration and invasion. Meanwhile, GA induced dose-dependent apoptosis of ESCC cells, repressed the expression of Bcl2 and up-regulated the levels of Bax protein, cleaved-PARP1 and cleaved-caspase 3/9. Further investigation showed that GA down-regulated the levels of PI3K, p-AKT and p-mTOR, while promoted PTEN expression in ESCC cells. Taken together, we provided the first demonstration that GA exerts anti-tumor effects on ESCC cells presumably through regulating PTEN-PI3K-AKT-mTORpathway, suggestive of a therapeutic potential for ESCC.

## Introduction

Esophageal squamous cell carcinoma (ESCC) is one of the most common malignancies with a low five-year survival rate, ranking sixth worldwide as well as third in China according to the incidence rate 1,2. Many breakthroughs in the study of esophageal cancer have been made, and surgery remains the best treatment option for this malignancy. Given that the chance of the cancer recurrence after surgical resection is still very high 3, developing new effective chemotherapy drugs is indispensable for the treatment of esophageal cancer.

Gambogic acid (GA), the main active component with a cage structure secreted by Garcinia Cambogia, shows remarkable anticancer activity in various human tumors, such as lung, gastric, hepatocellular, brain and colon cancer 4-8. In this case, GA inhibits cancer cell proliferation, induces apoptosis and suppresses tumor metastasis. So far, the anti-tumor effect of GA on ESCC has yet to be determined. Here, we aimed to identify the roles of GA in ESCC cell growth and apoptosis, and to explore the underlying mechanisms. This study could provide valuable data for elucidating the biological significance and therapeutic function of GA in ESCC.

## Material and Methods Reagents

Reagents, antibodies and kits in the study were purchased as follows: GA from Sellck (Houston, USA), antibodies for cleaved-caspase 3, cleaved-caspase 9, cleaved-PARP1, Bcl-2, MMP2, MMP9 and β-actin from Proteintech (Wuhan, China) antibodies for Bax, PI3K, m-TOR, p-mTOR (ser2448), cyclinB1, cyclinD1, totalAKT and p-AKT (ser473) from Cell Signaling Technology (Danvers, MA, USA), FITC-conjugated Annexin V kit for apoptosis detection from NEOBIOSCINECE (Shenzhen, China), 4',6-diamidino-2-phenylindole (DAPI) from Beyotime (Shanghai, China).

### Cell culture

YES2, KYSE30, KYSE150 and KYSE450 cell lines were obtained from the laboratory of Prof. Yutaka Shimada at Kyoto University in Japan. The ESCC cells were grown in RPMI 1640 medium containing 10% FBS, 100 U/mL penicillin and 100 μg/mL streptomycin in a 37 °C incubator with humidified 5% CO2.

### Cell viability assay

MTS assay was conducted to assess the cell survival rate. The cells at logarithmic growth phase were collected and uniformly seeded into three 96-well plates (3×103 cells/well). After 24 hours incubation, the cells were treated with 0, 0.5, 1 or 2 μM GA (6 sub-holes per concentration), followed by incubation for 24, 48, and 72 hours, respectively. 10 μL of MTS was added into each well with 90 μL of medium, and the cells were incubated for 1 hour in the dark. Finally, a microplate reader was used to measure the absorbance at a wavelength of 490 nm, and the cell inhibition rate was calculated according to the following formula: the inhibition rate (%) = (1 - absorbance value in the treated group / that in the control one) ×100%. Each experiment was conducted in triplicate, and the average value was taken as the experimental results.

### Colony formation assay

For this assay, logarithmically growing ESCC cells were uniformly inoculated into three 6-well plates. After 24 hours incubation, 0,0.5 or 1 μM GA were added into the plates, respectively, and the cells were grown for 12 hours. Then, the cells were harvested by centrifugation, and 2×103 cells in the normal medium were sub-cultured in a six-well plate. After 14 days, the surviving colonies were fixed in 100% methanol and stained using 1% crystal violet for 10 minutes. Finally, the plate was washed three times with running tap water and air dried at RT.

### Cell proliferation assay

The xCELLigence System Real-Time Cell Analyzer (RTCA-MP) (Roche Applied Science, ACEA Biosciences Inc, USA) was used to measure the rate of cell proliferation based on the size of each pore resistance 9,10.The logarithmically growing cells were uniformly inoculated into 96-well plates (3×103 cells in 100 μL culture medium per well). After 24 hours incubation, the cells were treated with 0, 0.5, 1, 2 or 4 μM GA. Electrical impedance-based proliferation assay was performed using RTCA system. The electrical impedance was recorded every 15 minutes and the rate of cell proliferation was expressed as a cell index (CI) ± SD.

### Cell apoptosis

Flow cytometry was conducted for apoptosis detection using the Annexin V-FITC kit (NEOBIOSCINECE, Shenzhen, China). Briefly, the ESCC cells were cultured with 0,0.5or 1 μM GA for 48 hours, and then subjected to a Leica DMI4000B microscope (Leica Microsystems GmbH, Wetzlar, Germany) for a morphological check. The attached and floating cells were treated with trypsin, followed by incubation with 5 μL of FITC-conjugated annexin V (0.5 mg / mL) for 15 minutes, and then 5 μL of PI (0.5 mg/mL) for another 15 minutes in the dark at RT. A BDTM LSRΙΙ flow cytometer (BD Biosciences, NJ, USA) was used to detect annexin V-positive cells in the samples that were recognized as apoptotic cells.

### TUNEL assay

The one step TUNEL kit was used to perform apoptosis assay as instructed by the manufacturer (Beyotime, Shanghai, China). Logarithmically growing ESCC cells were digested and centrifuged for 24 hours in a glass plate on a glass bottom cell culture dish (NEST, Wuxi, China). After treatment with 0, 0.5 or 1 μM GA for 24 hours, the cells were fixed in 100% methanoland incubated with 0.3% Triton X-100 for 5 minutes on ice for permeabilization. Cell samples were then resuspended in TUNEL reaction mixture, followed by incubation for 60 minutes at 37 °C. The green fluorescence at 530 nm detected in the apoptotic nuclei was observed by a Nikon fluorescence microscopy.

### Transwell assay

Logarithmic growth of the ESCC cells was conducted in a 6-well plate. After 24 hours culture, different concentrations of GA (0, 0.5 and 1μM)were added into the growing cells. The ESCC cells continued to grow for 12 hours, and were then digested and counted. 1×106 viable cells were resuspended in serum-free medium and uniformly seeded into the upper chambers of a Transwell plate. Subsequently, 600 μL of culture medium with 30% FBS was applied to each lower chamber. For an invasion assay, the upper chambers of the plates were pre-coated with 2% Matrigel at 37 °C for no less than 6 hours. The resuspended cells were directly applied to the pre-coated upper chamber with 50 μL of serum-free medium. After incubation for 24 hours, the remaining cells were immersed with pre-cold methanol for 10 minutes, followed by staining with 0.5% crystal violet for 10 minutes. The invading cells were photographed and quantified under a microscope.

### Western blotting

KYSE150 and KYSE450 cells were incubated with various concentrations of GA (0, 0.5 or 1 μM) for 24 hours, respectively. After washing twice with PBS, the cells were collected by trypsinization and centrifugation, and then subjected to a total protein extraction. Protein concentrations were measured by using Plyle BCA protein quantitation kit (Applygen, China). The equal amount of protein samples were separated by SDS-PAGE gel electrophoresis and transferred onto a PVDF membrane (Millipore, USA). The PVDF membrane was first blocked in 5% skim milk or 2% BSA and then incubated with primary antibodies, followed by the corresponding secondary antibodies (Abbkine, Wuhan, China). The protein targets in the membrane were detected and visualized by using the Chemiluminescence Luminol Kit. The following primary antibodies were applied in the experiments: cleaved-caspase3, cleaved-caspase9, cleaved-PARP1, phosphorylated AKT, Bcl-2, MMP2, MMP9 and β-actin (Proteintech Group, Wuhan, China); PARP1, BAX, PI3K, total AKT, phosphorylated AKT (ser473), p-mTOR (ser2448), cyclinB1 and cyclinD1 (Cell Signaling Technology, MA, USA). β-actin was chosen as a loading control in all experiments.

### Statistical analysis

Student's t-test was employed to calculate the difference between two groups based on GraphPad Prism 7.0 (GraphPad Software, San Diego, CA, USA). All experiments in the bar chart were carried out at least three times, and all samples were analyzed in triplicate. (SD). Statistically, *P*<0.05 was defined as significant.

## Results

### An inhibitory effect of GA on ESCC cell proliferation

**Figure 1:**
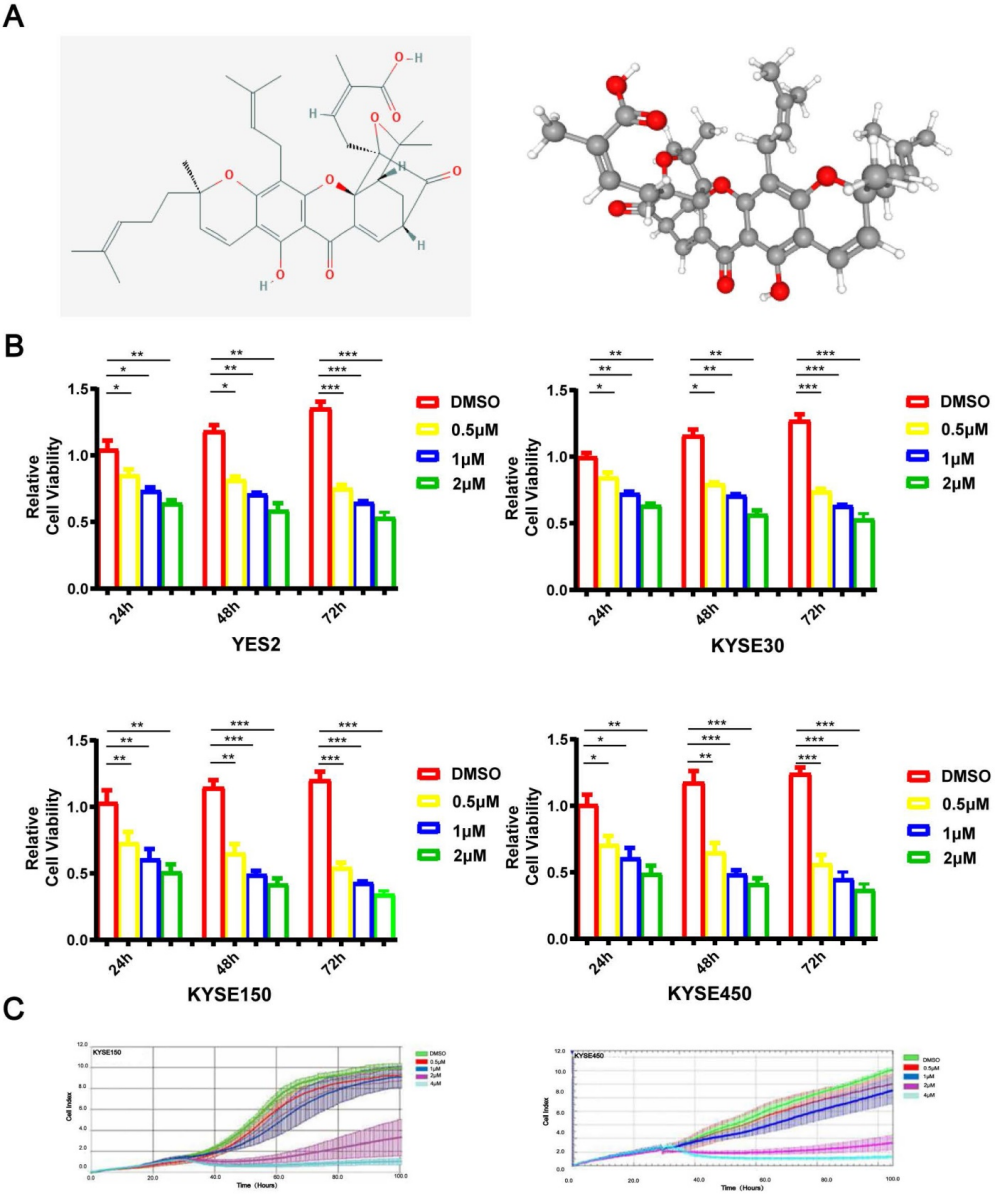
** GA attenuates ESCC cell proliferation. (A)** The chemical composition and 3D Conformerstructure of GA. **(B)** YES2, KYSE30, KYSE150 and KYSE410 cells were incubated with various concentrations of GA for 24 h, 48 h and 72 h. **(C)** Law determination. XCELLigence-RTCA-MP was used to detect the proliferative capacity of KYSE150 and KYSE450 cells administered with GA (0, 0.5, 1, 2 and 4 µM). Effects of GA on ESCC cell viability. *P<0.05, **P<0.01, ***P<0.001 for comparison between the experimental and control groups.

As depicted in Figure 1A, GA is a natural xanthone with remarkable anticancer activity in various human tumors. To investigate the role of GA in the proliferation of ESCC cells, YES2, KYSE30, KYSE150 or KYSE450 cell lines were administered with 0, 0.5, 1 or 2 μM GA, respectively. After incubation for 24, 48 or 72 hours, the cells were tested for the viability by using microscopy as well as MTS assays. As shown in Figure 1B, ESCC cells treated with GA exhibited morphological changes, and GA significantly inhibited the cell growth in two ESCC cell lines in a dose- and time-dependent manner. We next employed the RTCA-MP system to analyze the rate of cell proliferation in ESCC cells treated respectively with 0, 0.5, 1, 2 or 4 μM GA. Likewise, RTCA-MP system-based analysis revealed a dose-and time-dependent inhibitory effect of GA on cell proliferation in KYSE150 and KYSE450 cell lines (Figure 1C). Collectively, GA possesses a dose- and time-dependent inhibitory role in ESCC cell proliferation.

### GA inhibits cell migration and invasion in ESCC

**Figure 2:**
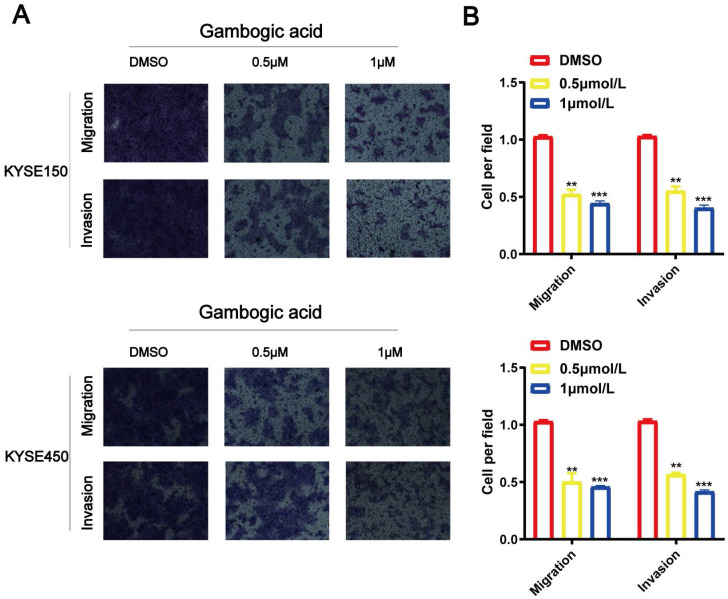
** GA suppresses ESCC cell migration and invasion. Effects of various doses of GA on ESCC migration were determined using Transwell assay. (A, C)** GA treatment reduced the invasion and migration capacity of KYSE150 and KYSE450 cells in a dose-dependent manner. **(B, D)** Bar chart of number of migrating and invading cells; *P<0.05, **P<0.01, ***P<0.001 for comparison between the experimental and control groups.

Transwell assays for KYSE150 and KYSE450 cell lines were performed to identify biological function of GA in the cancer cell migration and invasion. As shown in Figure 2, the number of migrated and invaded cells were significantly reduced in the ESCC cell lines administered with various concentrations of GA compared with the control groups. The experimental results demonstrated that GA could reduce the invasion and migration ability of two esophageal cancer cells.

### GA inhibits ESCC colony formation

**Figure 3:**
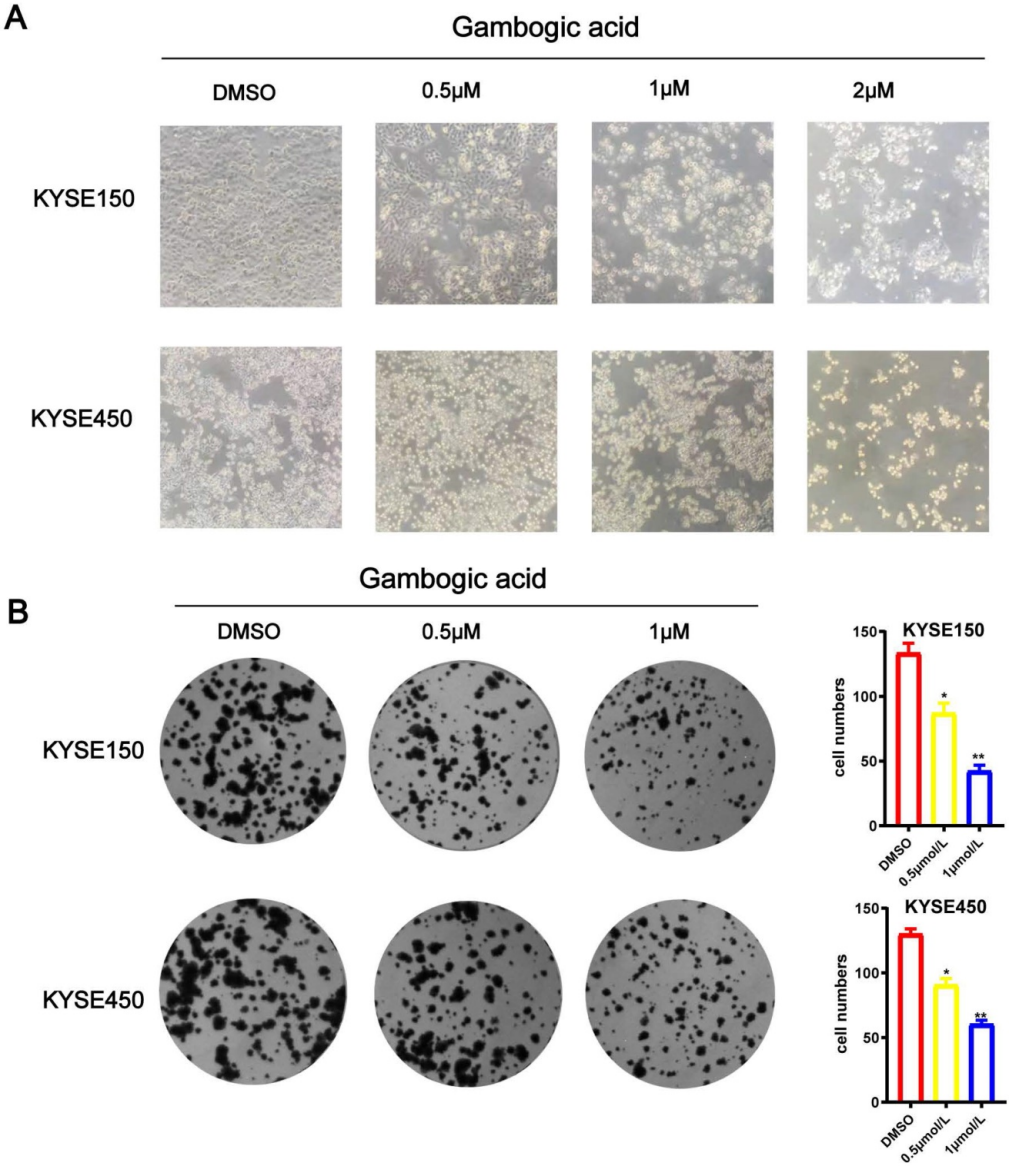
** GA inhibits ESCC cell colony formation. (A)** Morphology and number of the ESCC cells incubated with GA (0, 0.5, 1 and 2 µM) for 24 hours were examined by microscope. **(B)** KYSE450 cell clone formation ability. GA treatment led to a dose-dependent decrease in colony formation of the ESCC cells. *P<0.05, ***P<0.001 for comparison between the experimental and control groups.

We treated esophageal cancer cells with different concentrations of GA 0, 0.5 or 1 μM for 24 hours, and observed the morphology of KYSE150 and KYSE450 cells with the microscope. As the GA concentration increased, the number of esophageal cancer cells decreased, the volume became rounder and smaller, the cytoplasm concentrated, the membrane disintegrated, some of the nuclei destroyed and disappeared, moreover, the number of cells decreased. As depicted in Figure 3A. Meanwhile, colony formation assay revealed that the colony formation ability of the ESCC cells significantly decreased as GA concentrations increased (*P* < 0.05) (Figure 3B).Together, these data demonstrated that GA treatment attenuated the ability of ESCC cells in colony formation and changed the cell morphology in a concentration-dependent manner.

### GA induces ESCC cell apoptosis

**Figure 4:**
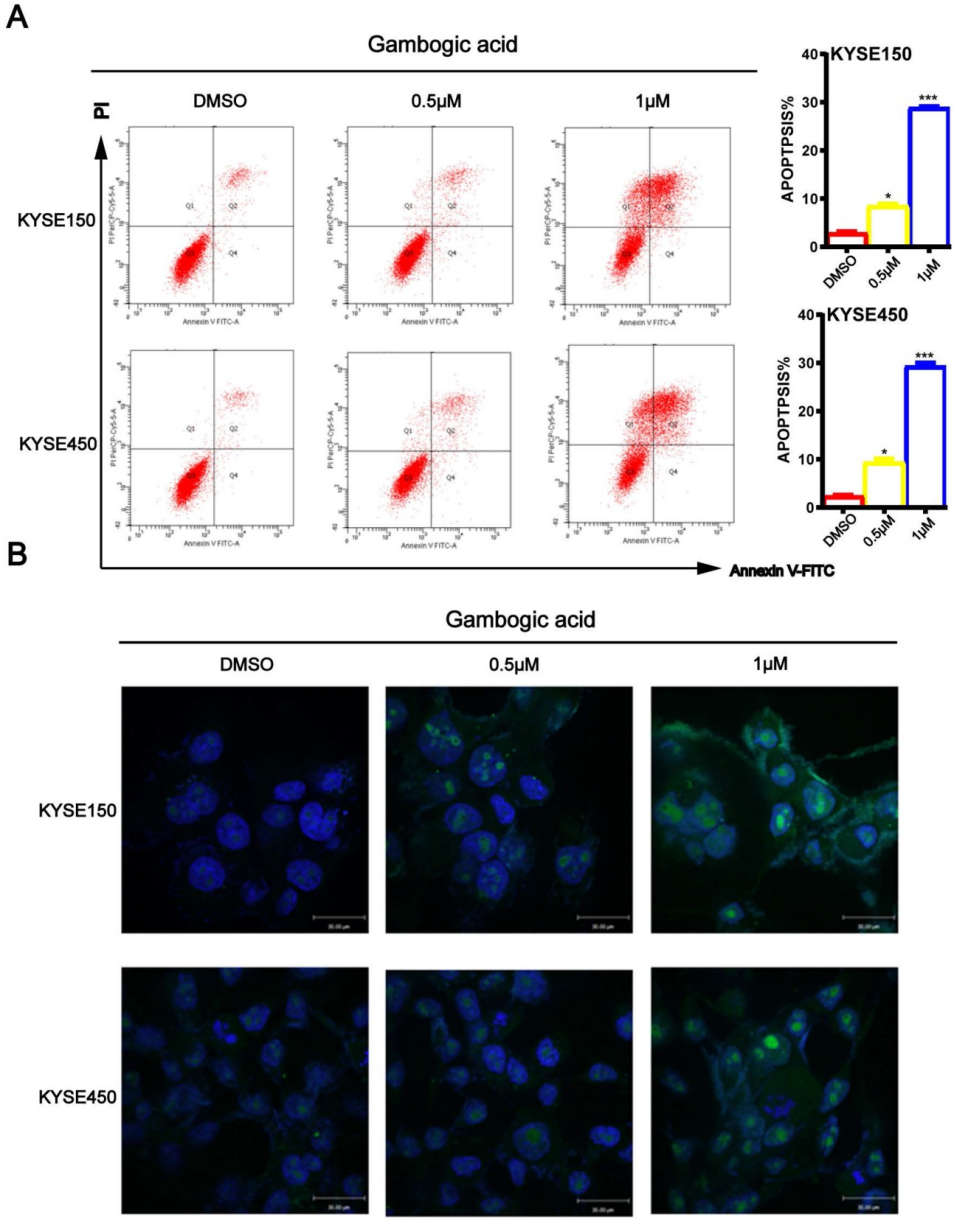
** GA promotes apoptosis in ESCC cells. (A)** Immunofluorescence assay and flow cytometry were performed to assess apoptosis. **(B)** TUNEL assay based detection of apoptosis in the cancer cells incubated with GA (0, 0.5 and 1 µM). Morphological examination on cell nucleus was conducted by immunofluorescence assay. Representative data and the proportion of apoptotic cells were indicated respectively. *P<0.05, ***P<0.001 for comparison between the experimental and control groups.

To determine whether GA induces ESCC cell apoptosis, KYSE150 and KYSE450 cells treated with GA were double-stained with Annexin V/PI, and then subjected to the flow cytometry. As shown in Figure 4A, increasing concentrations of GA resulted in significantly higher number of apoptotic cells in the two ESCC cell lines compared with the control groups. Next, we performed TUNEL assays to detect apoptosis in the two cell lines 24 hours after treatment with GA. In accord with the findings on flow cytometry, the TUNEL assay revealed that the two ESCC cell lines harbored more staining positive cells than the control groups (Figure 4B). On the contrary, 0.5 μM and 1 μM GA had little effect on the cell cycle of treated cells (Figure S1), as shown in the above observations.

### GA treatment elicits an apoptosis in ESCC cells

**Figure 5:**
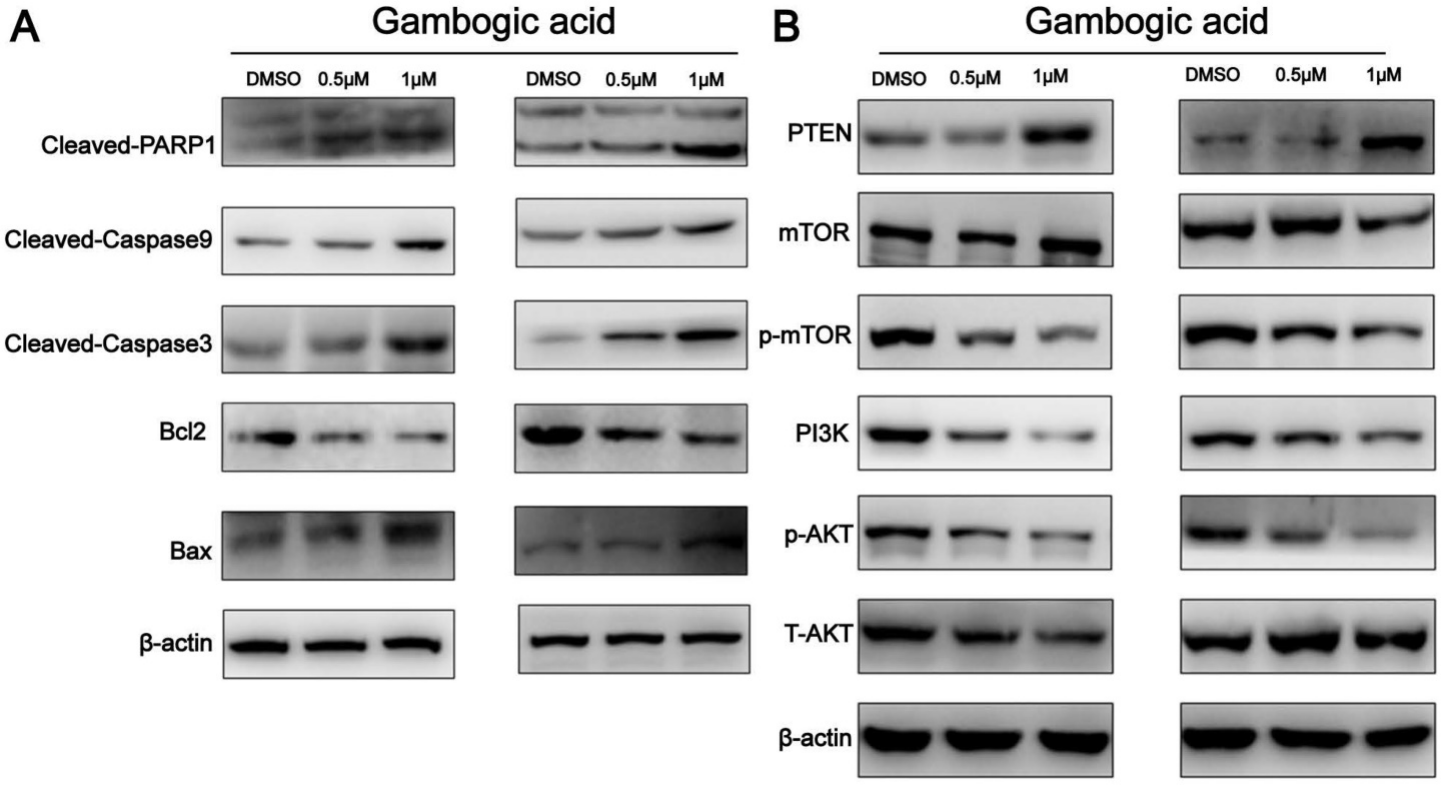
** Expression of apoptosis-related proteins and PI3K/AKT/mTOR pathway components in ESCC cells incubated with GA. (A)** The expression levels of cleaved-PARP1, Bcl-2, Bax, cleaved-caspase 3 and cleaved-caspase9 were detected by using western blot analysis. **(B)** Western blotting was performed to analyze the levels of PI3K, p-AKT, p-mTOR, AKT, mTOR and PTEN.

To further characterize GA-induced apoptosis, we analyzed the expression levels of Bax and Bcl-2 in the two ESCC cell lines by western blot analysis. As shown in Figure 5A, GA treatment led to a decrease in Bcl-2 expression and an increase in the expression of Bax protein in the cells, causing an elevated Bax/Bcl-2 ratio. Moreover, western blot revealed the increased levels of cleaved-Caspase3, cleaved-Caspase9 and cleaved-PARP1, but decreased PARP1 in the cells (Figure 5A). These findings suggest that GA-induced apoptosis in ESCC cells involves the caspase-dependent pathway.

### GA may down-regulate PI3K/AKT/mTOR pathway through activating PTEN

It has been shown that PI3K/AKT/mTOR signaling pathway is important in controlling proliferation and apoptosis of various cancer cells 11. PTEN was found to act as a key upstream inhibitor of PI3K/AKT signaling 12. To determine whether PI3K/AKT/mTOR signaling and PTEN are involved in GA mediated functions in ESCC, we examined the expression of related proteins in the cancer cells treated with GA. As shown in Figure 5B, western blot analysis revealed that GA treatment for 24 hours led to a dose-dependent reduction in the levels of PI3K, p-AKT and p-mTOR, whereas an increase in the expression of PTEN in KYSE150 and KYSE450 cells. In this case, 0.5 μM and 1 μM GA displayed an inhibitory effect on the expression of PI3K, p-AKT and p-mTOR. Notably, we observed that the levels of p-AKT and p-mTOR were down-regulated in the cancer cells treated with 0.5 and 1 μM GA for 24 hours, while the expression levels of AKT and mTOR remained unchanged. Likewise, we detected a reduction in the expression of proteins related to PI3K/AKT/mTOR pathway in the cancer cells treated with 0.5 μM GA for 24 hours or 48 hours (Figure S2). Taken together, all these findings suggest that PTEN activation and inhibition of PI3K/AKT/mTOR signaling pathway may be involved in GA mediated functions in ESCC cells.

## Discussion

Esophageal cancer is a malignancy with a relatively low five-year survival rate, and clinical treatments are especially important for the management of this disease. Despite the enormous progress in therapeutic methods, the improvements in ESCC prognosis are still limited 13. Given that ESCC does not respond to the most conventional chemotherapeutic agents 14,15, developing new drugs for chemotherapy will be beneficial for ESCC treatment. In this case, natural small molecule drugs have been shown to have this potential due to the fact that they are less toxic and do not cause unwanted side effects 16. Here, we showed that GA suppressed the proliferation, invasion and migration of ESCC cells. Furthermore, GA may exert the antitumor effect on ESCC via the induction of apoptosis, as indicated by reduced expression of Bcl2 and PARP1as well as increased levels of Bax, cleaved PARP1 and cleaved-caspase3/9 in the cancer cells treated with GA. Moreover, molecular mechanistic studies suggest that down-regulation of PI3K/AkT/mTOR pathway may be involved in GA mediated antitumor effects on ESCC cells.

As a natural ingredient of herbs, GA possesses anti-inflammatory, anti-microbial and anti-tumor activities 17-19. In the present study, we observed a potent antitumor effect of GA on esophageal cancer, providing more data that GA suppresses various human cancers 7,20-23.Tumor metastasis occurs as cancer cells grow away from the original site, migrate through the blood or lymph system, and form new tumors in other sites. Metastasis is closely related to cancer progression 24, and cancer metastases, rather than the primary cancer, accounts for up to 90% of cancer deaths 25,26. Based on transwell, xCELLigence RTCA-MP and MTS assays, we showed that GA exhibits a remarkable anti-proliferative effect on ESCC cells as well as a time- and dose-dependent inhibition in ESCC cell migration and invasion.

Metastasis of tumor cells turns out to be mainly responsible for the deaths of ESCC patients. According to relevant researches, tumor invasion and migration serves as a multi factor and multi-step biological process, which mainly involves cell adhesion, protein degradation and migration. Among them, adhesion is deemed as the initial cause of cell development. Based on our study, it was found that with the increase of GA concentration, the number of esophageal cancer cells decreased, their volume became smaller and rounder, and the cytoplasm concentrated. Meanwhile, their adhesion, invasion and migration ability decreased as well. In the process of tumor metastasis, tumor cells pass through the extracellular matrix, thus realizing the migration and invasion of tumor cells, which serves as a significant part of the whole migration and invasion process. Metalloproteinases (MMPs) play a critical role in promoting tumor cell migration, extracellular matrix degradation and tumor distant metastasis, among them MMP2 and MMP9 are closely related to the proliferation, invasion and metastasis of ESCC. In this study, the effects of GA on the expression of MMP2 and MMP9 in ESCC cells were supplemented. When treated with GA, the expression of MMP2 and MMP9 in ESCC cells decreased. It is demonstrated in results of molecular experiments that GA can significantly inhibit the expression of MMP2 and MMP9 in ESCC, which further proves that GA may inhibit the invasion and migration of MMP2 and MMP9 by changing cell morphology to reduce adhesion, as well as inhibiting the expression of MMP2 and MMP9 in ESCC (Figure S3).

As a promising target for anticancer therapy, apoptosis has been used in screening and evaluating the chemotherapy drugs. It is a form of programmed cell death that is highly regulated and genetically controlled process for maintaining homeostasis 27,28. In this study, we observed a GA-induced apoptosis in the ESCC cells. Tunel assay and flow cytometry were employed to assess the effect of GA on the cell death in the cancer cells. Compared with the control groups, cells treated with GA presented distinct morphological changes, such as cell volume contraction and chromatin condensation. Furthermore, GA treatment led to a significant dose-dependent increase in the number of cells in the early or late stages of apoptosis. The Bcl-2 protein family, including anti-apoptotic and pro-apoptotic members, are significant regulators of mitochondrial function. As an anti-apoptotic protein, Bcl-2 inhibits apoptosis, while Bax acts as pro-apoptotic protein, promoting apoptosis. Thus, the Bcl-2/Bax ratio is important for cell survival or death 29. Increased ratio of Bax/Bcl-2 leads to a release of cytochrome c from mitochondria that activates Caspase3 and Caspase9. Activated Caspase3 and Caspase9 subsequently cleave the substrates such as PARP, thereby inducing apoptosis 30,31. Here, we found that GA treatment up-regulated cleaved Caspase3, cleaved Caspase9 and cleaved PARP1, while down-regulated Bcl-2. In the meantime, western blot analysis revealed an increased ratio of Bax/Bcl-2 in the cancer cells treated with GA. Thus, we propose that GA elicits ESCC cell apoptosis via the mitochondria-dependent apoptotic pathway.

Tumors are a type of disorders characterized by uncontrolled cell growth. Abnormalities in cell proliferation, metastasis, and apoptosis are involved in tumorigenesis and development. Altered cell cycle is considered the most important mechanism of tumors. Multiple studies have shown that anti-cancer Chinese medicine can block tumor cells at different cell cycle stages. It is known that many factors, such as cell cycle dependent kinases, cell cycle inhibitory proteins and signal pathway components, function in the cell cycle arrest. Thus, in-depth study of the above factors could lead to an identification of the targets of traditional Chinese medicine on cell cycle control, and lay a solid foundation for better clinical application of traditional Chinese medicine. In the present study, we analyzed the cell cycle distribution in the ESCC cells treated with GA by using flow cytometry and western blotting. The results showed that 0.5 μM or 1 μM GA had little effect on the ESCC cell cycle. Based on these observations, we mainly focused on GA induced apoptosis as well as the underlying signaling pathways.

PI3K/AKT/mTOR signaling pathway has a pivotal role in malignancies 32-35. Recent studies have shown that dysfunction in PTEN/PIK3/AKT/mTOR signaling is involved in tumorigenesis 36. As an important tumor suppressor gene, PTEN negatively regulates PI3K/AKT/mTOR pathway, inhibiting cell growth and proliferation 37,38. The findings in this study suggest that GA attenuates ESCC cell growth presumably through regulating PTEN and PI3K/AKT/mTOR pathway.

In conclusion, this study provides the first demonstration that GA exerts the anti-tumor effects through inducing apoptosis and inhibiting PI3K/AKT/mTOR pathway.

## Supplementary Material

Supplementary figures.
