# Supplementary material for: Gambogic acid affects ESCC progression through regulation of PI3K/AKT/mTOR signal pathway
Source: J Cancer. 2020 Jul 20;11(19):5568–77. doi: 10.7150/jca.41115 (PMC7477450; doi:10.7150/jca.41115)
Supplement: Supplementary file 1 — Supplementary figures. [file jcav11p5568s1.pdf]

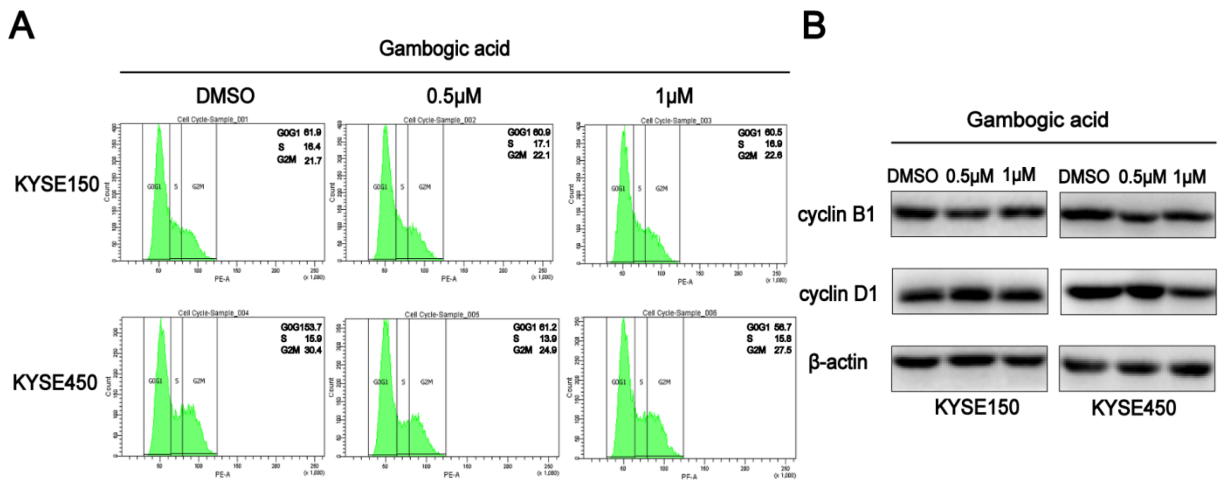

Figure S1. GA has little effect on the ESCC cell cycle control. **(A).** Representative data on the cell cycle detection. **(B).** CyclinB1 and cyclinD1 were detected by using western blot analysis.

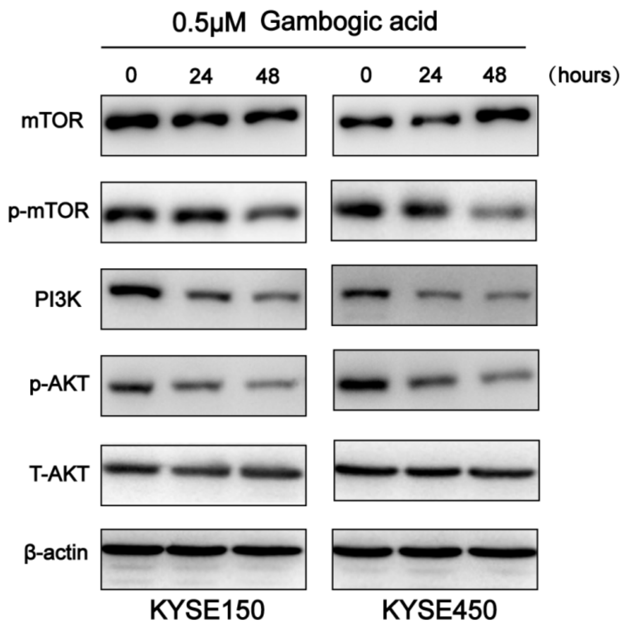

Figure S2.GA negatively regulates PI3K/AKT/mTOR pathway at different times.Western blot was conducted to assess the expression of PI3K, p-AKT, p-mTOR, AKT and mTOR.

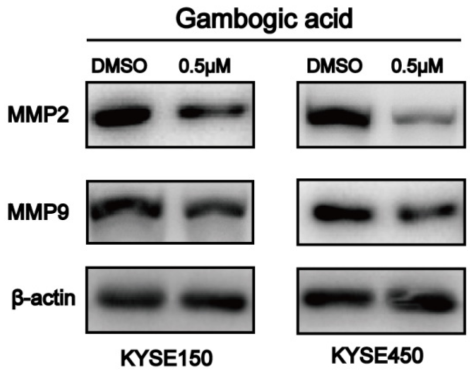

Figure S3.GA treatment of ESCC inhibited the expression of MMP2 and MMP9. Western blot detected the expression of MMP2 and MMP9.
